# Family UNited: piloting of a new universal UNODC family skills programme to improve child mental health, resilience and parenting skills in Indonesia and Bangladesh

**DOI:** 10.1186/s13033-023-00602-w

**Published:** 2023-12-11

**Authors:** Karin Haar, Aala El-Khani, Narendra Narotama, Amir Hussain, Eva Fitri, Aip Badrujaman, Eka Wahyuni, Shah Mohammad Naheeaan, Ali Yassine, Wadih Maalouf

**Affiliations:** 1 Prevention, Treatment and Rehabilitation Section (PTRS), Drugs, Laboratory and Scientific Services Branch (DLSSB), Division for Policy Analysis and Public Affairs (DPA), United Nations Office on Drugs and Crime (UNODC), Wagramer Strasse 5, 1400 Vienna, Austria; 2 Division of Psychology and Mental Health, University of Manchester, Manchester, England; 3 Drug Demand Reduction Division, United Nations Office on Drugs and Crime (UNODC), Menara Thamrin Building 10th Floor, Central Jakarta, 10250 Indonesia; 4 Clinical Psychologist, Nasirullah Psychotherapy Unit (NPU), Dhaka Clinical Psychology Department, University of Dhaka, Room 5017, Arts Building, Dhaka, Bangladesh; 5 UN certified External Stress Counselor United Nations Department for Safety and Security (UNDSS), Dhaka, Bangladesh; 6 Drugs Prevention Campaigner, Advocacy Directorat at Deputy of Prevention, National Narcotics Board of Indonesia, Jakarta, Indonesia; 7 Guidance and Counselling Department, State University of Jakarta, Jakarta, Indonesia; 8 United Nations Office on Drugs and Crime (UNODC), Dhaka, Bangladesh

**Keywords:** Universal family skills programme, Child mental health, Child resilience, Parenting skills, Indonesia, Bangladesh

## Abstract

**Background:**

Family is one of the most influential social institutions and caregivers act as the main protective factors for children’s mental health and resilience skills. Family skills programmes support caregivers to be better parents and strengthen positive age-specific and age-appropriate family functioning and interactions. We developed a universal, brief and light programme for implementation in low-resource settings, the Family UNited (FU) programme, and conducted a pilot study to show feasibility of implementation, replicability and effectiveness in improving family functioning, child behaviour and resilience.

**Methods:**

We recruited caregivers with children aged 8–14 years through schools in East Java, Indonesia and Dhaka, Bangladesh to the FU programme. Demographic data, emotional and behavioural difficulties of children, child resilience and parental skills and family adjustment measures were collected from children and caregivers before, 2 and 6 weeks after the intervention. Outcome was assessed through the SDQ (Strengths and Difficulties Questionnaire), PAFAS (Parenting and Family Adjustment Scales) and CYRM-R (Child and Youth Resilience Measure).

**Results:**

We enrolled 29 families in Bangladesh and allocated 37 families to the intervention and 33 to the control group in Indonesia. Overall, there was no effect over time in the control group on any of the PAFAS subscales, whereas significant reductions in scores were found on six of the seven subscales in either country in the intervention group, most prominently in caregivers with higher scores at baseline. We found highly significant reductions in total SDQ scores in the intervention group in both countries, whereas there was no effect over time in the control group in Indonesia. Boys in the intervention group in Indonesia and in Bangladesh seemed to have benefitted significantly on the SDQ as well as the total resilience scale. Overall, on the CYRM-R, particularly children below the 33rd percentile at pre-test benefitted substantially from the programme.

**Conclusions:**

The implementation of a brief family skills programme was seemingly effective and feasible in resource-limited settings and positively improved child mental health, resilience and parenting practices and family adjustment skills. These results suggest the value of such a programme and call for further validation through other methods of impact assessment and outcome evaluation.

*Trial registration*: Clinical Trial Registration: ISRCTN99645405, retrospectively registered, 22 September, 2022.

## Background

A significant part of the available research shows that poor parenting has a negative impact on the physical [1], mental and behavioural well-being of children [2]. For example, low levels of warm involvement, physical aggression and inconsistent and coercive parenting were found to significantly cause problematic behaviour in children as well as negative mental health outcomes, such as declined school performance or even failure, drug use, increased violence [3] and more. Moreover, parental mental well-being, family cohesion and positive parent–child relationship are some of the protective factors for children’s mental health, whereas poor parental mental health, parental substance abuse and assertive family relationships are risk factors for children’s mental health [4] and carry an impact on child’s development [5].

Parenting characteristics and family functioning carry also an impact on children’s overall resilience. In the past, resilience has been described as the capacity to bounce back or cope successfully despite substantial life challenges or in the face of stress or adversity [6, 7], and is more likely to develop for children who are brought up in a positive and supportive family environment [8]. Resilience reflects the capacity of an individual to reach out for and make use of meaningful socioecological resources that support his or her well-being and healthy development in times of stress [9]. Caregiver support has the same positive impact on child resilience as it does on adolescent resilience. In a study by Haar et al. evaluating the impact of a family skills intervention, children showed a significant improvement both physically and psychosocially post-intervention, including in terms of mental health and resilience [10]. Furthermore, research on child resilience through family skills support reflects its protective factor against many negative outcomes, including violent extremism [11].

Family functioning and parenting characteristics are nevertheless under the influence of many social and environmental factors. Given that in low and middle-income countries, detrimental macro-level negative influences are more prominent, children and families in these contexts have an imminent need of support. [12–16]. Nevertheless, the development and evaluation of family skills programmes has been rather more concentrated in high-income countries which accentuated the gap in transferability and applicability of such tools to countries of low and middle income.

Research focusing on the parent–child relationship dynamics is crucial to understand the etiology of problematic behaviour in children. Furthermore, the inclusion of under-researched contexts (such as low- and middle-income countries in this case) is crucial for the development, adaptation and adjustment of suitable prevention practices and responses, such as family skills programmes that aim to enhance parenting skills.

### Family skills programmes in general and UNODC’s work

Family skills programmes provide a range of parenting knowledge, skill building, competency enhancement and support [17]. They aim to strengthen family protective factors such as communication, trust, problem-solving skills and conflict resolution, and strengthen the bonding and attachment between caregivers and children. These skills allow parents to cope and adapt to the different challenges that arise with parenting children. They promote a warm child-rearing style where parents set rules for acceptable behaviours, closely monitor free time and friendship patterns and become good role models while helping their children to acquire skills to make informed decisions. Such skills and family functioning have been reflected to prevent several negative social outcomes, including drug use, school drop-out, child maltreatment, mental health adversity and childhood aggression [18].

Nevertheless, most research on such outcomes have been documented in high-income and stable context countries with fewer studies emanating from families living in Low- and Middle-income countries [19, 20]. Some of the recent research exceptions included a pilot randomized controlled trial (RCT) of a parenting intervention with war-affected caregivers in Lebanon, reporting positive impacts on parental stress and discipline practices [21]. A second study describing the implementation of a family skills intervention with Syrian refugees indicated increased parental warmth and responsiveness, decreased harsh parenting, lowered stress and distress, improved psychosocial wellbeing among the intervention group [22]. A third study assessing the effectiveness and acceptability of a parenting intervention among 292 Syrian refugee parents and 88 of their children in Lebanon, showed promise in reducing child maltreatment and improving child and parental mental health in a humanitarian setting [23]. Such research, on one end, supports the transferability of such science to such settings and on the other incite for stretching the availability of such tools for families in more challenging contexts. A recent article further emphasised a significant lack of family skills interventions being utilised in humanitarian settings compared to global implementation and made recommendations for this area to be urgently developed [20]. Inspired by this body of knowledge, UNODC Prevention Treatment and Rehabilitation Section has been actively promoting and piloting such evidence-based programmes globally since 2010 in over 30 low- and middle-income countries.

### Family United aims, experiences and targeted settings

In 2018, UNODC developed a family skills programme called ‘Strong Families’, which was tailored for challenged and humanitarian settings (selective level prevention). Building on the positive evidence emerging from Strong Families [10, 24, 25], UNODC developed a new family skills programme called “Family UNited” [26]. Family UNited was designed for implementation in wider settings (universal level prevention) accounting for families living in low- and middle-income country context. [27, 28]

The Family UNited programme was drawn from three overarching theories which shaped the components of the programme sessions. Firstly, the Biopsychosocial Vulnerability Model [29] which suggests that positive family coping skills such as conflict resolution, active problem-solving skills and positive communication, shield individual family members and protect youths’ vulnerability from the negative effects of family conflicts. In this theory, caregivers influence on their children is greatest when the children are younger and decreases significantly as they enter early adolescence. The second theory is the Resiliency Model [30], which emphasizes the foundational role caregivers in a family play in children developing resilience. Resilience is defined as the ability to rebound from difficult or adverse circumstances [31] and is thought to more likely develop for children when raised in a family environment in which caregivers are both positive and supportive [8]. This theory focuses on life skills that are promoted when caregivers are supportive, such as reflective skills, emotional management skills and the ability to problem solve. This theory is supported by research that identifies that the relationship a child has with their caregiver can have a more significant impact on their mental health projectory than from the experiences of war and displacement [32]. The third theory is Social Learning Theory [33] which proposes that children’s daily experiences of the world through their interactions with others, imitation, and the reinforcement they receive, shapes their behaviour both directly and indirectly [34]. This places the role of caregivers as pivotal for their healthy social developmental and also guides family skills interventions to focus on improving the quality of parenting by improving foundational parenting skills [35].

**Fig. 1:**
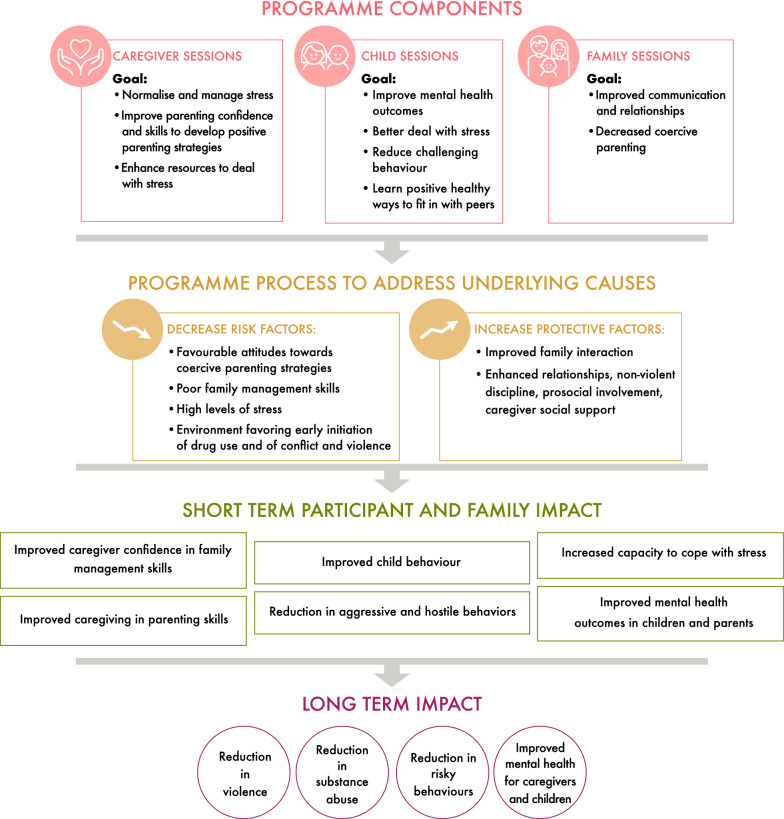
Logic model of the Family UNited programme

Based on its theoretical foundations, the content of the Family UNited Programme focuses on enhancing an empathetic and warm approach to caregiving; improving family cohesion, communication and relationships; and skills for emotional regulation and assertive skills for managing peer pressure, as outlined in the logic model (Fig. 1).

Within the caregiver sessions, caregivers learn how to normalize and manage stress and how to improve their parenting confidence and skills in order to develop positive parenting strategies. In parallel, also children learn on how to better deal with stress, how they can reduce challenging behaviour and children also learn positive and healthy ways on how to fit in with peers. The main aim of the family sessions is to improve the communication and relationships between children and their caregivers and to decrease coercive parenting behavious. Overall, Family UNited aims to decrease risk factors, such as poor family management skills, high levels of stress and an environment favoring early initiation of drug use and other risky behaviour and it increases protective factors, such as family interactions and relationships, non-violent discipline and prosocial involvement. On a short term, Family UNited aims to improve parenting skills, child behaviour and the capacity to cope with stress, whereas on the longer term it aims to reduce violence, substance use and risky behaviours and improve mental health for both, caregivers and children.

Given ongoing operations of UNODC global programme segment, Indonesia and Bangladesh are two lower middle-income countries selected for piloting Family UNited. Both countries with high population density and low infrastructure, making them suitable for the Family UNited programme pilot testing. Simultaneously, the request and facilitation of both Indonesian and Bengali governmental counterparts of the Family UNited programme implementation made both countries a priority choice for the piloting of the aforesaid programme.

### Bangladesh general context

Bangladesh has an estimated population of 161 million with the majority of the population being young: approximately 75 million are under 25 years old (27% 0–14 years and 38% 15–24 years). Among those, 46% are women (approximately 35 million). In recent times, drug use and risky behaviour have become some of the emerging problems in Bangladesh [36]. While the family is one of the most powerful social institutions in Bangladesh, several social and economic stressors carry negative effect on its functioning nationally. In Bangladesh the child mental health status reflects an imminent need for preventive action. In a National Mental Health Survey report the prevalence of mental health disorders among children was 13.6% in 7–17 year-olds in 2018/19. According to this survey, almost 95% of children diagnosed with mental disorders in Bangladesh did not get treatment for their condition. Similarly, 16.8% of adults reported mental health disorders [37], compared to 16.1% in the 2003–2005 survey [38]. Among all adults diagnosed with mental disorder, 92% did not receive treatment in 2018–19 [37].

### Indonesia general context

Indonesia is one of the largest economies in Southeast Asia and the world’s 10th largest in purchasing power parity due to rapid economic progress over the past twenty years. Indonesia is also managed to cut the poverty rate by more than half since 1999, to under 10% in 2020. Indonesia is also known as a diverse archipelago nation with more than 300 ethnic groups and the world’s fourth most populous country and a member of the G-20 [39]. Indonesia has shown remarkable progress over the past twenty years and has joined the ranks of the Middle-Income Countries (MICs) and emerged as the biggest economy in Southeast Asia.

In terms of the drug situation, Indonesia is not a major producer of illicit drugs but has long been targeted as a source and transit country by transnational organized criminal trafficking in heroin and cocaine [40]. Although drug use in Indonesia has long been dominated by cannabis, the late 1990s saw a substantial increase in heroin use. In more recent years, the country has also become a destination point for the trafficking of amphetamine-type stimulants, primarily ecstasy and crystalline methamphetamine. According to the results of a Population Survey 2020 by the Indonesia Central Statistic Agency, the majority of Indonesia’s population are young and in the economically productive age range. Mental health is an essential component for health and wellbeing of children. Mental health is considered as the biggest contributor to years of life lost due to premature mortality around 14.4% globally, 13.5% in Southeast Asia and 13.4% in Indonesia [41]. The number of mental health problems among children in Indonesia has increased over the years. It is estimated that mental health problems affected 10% of child population in Indonesia [42].

The above scenario represents a challenge for the government that advocates for prioritizing prevention activities targeting Indonesian youth in the context of substance use and mental health. Prevention programmes targeting families, has been a priority for the Indonesian National Narcotics Board (BNN). Nevertheless, such packages have been conducted with the Anti-drug insight development programme and not necessarily in line with the UNODC WHO International Standards on Drug Use Prevention [43]. Other local approaches conducted in prevention was through the strengthening of women’s role in one of women local organisations (PKK). Such programmes aims to train women as facilitators providing drug prevention information to family members through the engagement as a PKK’s cadre in the community [44].

Against the background of both countries, coupled with: (1) the presence of active UNODC offices supporting national programming; (2) UNODC ongoing programmatic approach focused on strengthening family skills implementation in line with the UNODC WHO International Standards on Drug Use Prevention, (3) lack of open sourced family skills packages designed and piloted in Low- and Middle Income Countries and (4) interest of local governmental authorities in both Bangladesh and Indonesia to engage with UNODC on such programme; UNODC initiated a pilot of a new family skills tool (Family UNited in both countries).

## Methods

### Aim of our study

The aim of this study was to assess the feasibility of implementation and the effectiveness of Family UNited against its logic model. Accordingly, we conducted a pilot study in Bangladesh and further included a comparison group to an intervention group in Indonesia to measure short-term changes. We measured child resilience as the increased capacity to cope with stress, child mental health and behaviour and parenting and family adjustment skills in caregivers.

### Programme intervention

Family UNited is a group intervention for children and their primary caregivers with sessions attended over 4 weeks (one session per week). Up to two parents or main caregivers attend with a maximum of two children under their care aged eight to 14 years. Caregivers and children attend group sessions with up to 12 other caregivers and children. Each week the same 12 caregivers attend the programme accompanied by their children for two hours. On arrival, children and caregivers from each family split into two separate rooms for the first hour and take part in group ‘child’ or ‘caregiver’ sessions. Then, during the immediate second hour, all families and facilitators group together in one room for the ‘family’ session.

The caregiver session in week one focuses on practicing strategies to increase their influence as a parent and working to understand how to praise and encourage children. Caregivers learn how attention changes behaviour and how to use reward, praise and give specific instructions. Children discuss and explore how to develop and practice positive qualities for themselves and begin to think of goals for their future. During the family sessions children and caregivers come together to discuss what positive qualities they would like for their family, the values they want to represent and and how to implement these in their family daily life. The caregiver session in week two focuses on encouraging good behaviour and discouraging misbehaviour and strategies to increase their influence as a parent. Caregivers practice skills of giving effective instructions and being clear about rules and expectations. They also learn about using appropriate consequences with their children. Children in week two explore what ‘stress’ means and begin to normalize feelings they may experience when stressed. They also learn stress management techniques. During the family session, caregivers and children come together and take part in activities that provide an opportunity to learn about each other, practicing positive communication and stress relief techniques together. In week three, caregiver practice using both love and limits in interacting with children and the importance of listening and communicating effectively. They learn how to match consequences to the actions of their children when responding to undesirable behaviour. This is achieved through role plays, interactive activities, and group discussions. Children are introduced to discussions on skills to resist peer pressure and during the family session, together with their caregivers they practice such skills through role play and group presentations, working further on building and developing family connections. In the final week, week four, caregivers learn and practice strategies to reduce and manage children’s aggressive behaviour through setting rules and discussing consequences. Children explore the meaning behind a ‘good friend’ and continue to practice skills to resist peer pressure. In the family session, families begin by peer pressure resistance practice between children and their caregivers, then take part in fun games that draw the skills they have been learning collectively together as they plan their goals for the future (Table 1).

**Table 1:** Overview of the structure and content of the Family UNited programme

	Caregiver session	Child session	Family session
Week 1	Understanding praising and encouraging children	Building positive qualities	Our family’s positive qualities
Week 2	Changing challenging behaviour	Handling stress	Learning about each other
Week 3	Responding to un-desirable behaviour	Skills to resist peer pressure I	Understanding peer pressure and family connections I
Week 4	Communicating and taking care of yourself	Skills to resist peer pressure II	Understanding peer pressure and family connections II

Induction of Family UNited prior to piloting was ensured by availing national inter-ministerial review of the Bangla translated materials (ensuring cultural suitability including for gender, age, and religion), followed by a political level advocacy meeting with different national counterparts as well as family skills describing the added value of the materials within the context of the UNODC WHO International Standards. A similar process was used for the Bahasa translation in Indonesia with the addition of focus group discussions with the researchers and family-based practitioners of BNN to ensure inclusivity and national ownership.

### Trial design, sampling, eligibility criteria and group allocation

In Bangladesh, we only included an intervention group, due to limited research resources. However, in an effort to contrast changes in a comparison group, we conducted a multisite non-blinded (researchers knowing which group is receiving intervention) controlled trial with two arms to assess effectiveness in Indonesia: (1) Intervention group: receiving the Family UNited programme and (2) Control group: Families completing only all data collection points. We prospectively collected outcome data assessing changes in parenting skills and family adjustment in caregivers, children’s behaviour, and children’s resilience capacities.

Participation in the study was done through a ‘universal’ approach, this generated an opportunistic sample, in which families with children aged eight to 14 years targeted participated based on interest and availability. This was deemed suitable for the purpose of the study objectives as the samples did not need to necessarily be representative of the Indonesian or Bangladeshi population but rather we needed to have 2 different cultures of families to corroborate if the programme effectiveness results are transferable. Inclusion criteria in the programme and the study included speaking Bangla/Bahasa, willing to take part in the programme and being in the town for the duration of the whole study and measurement meetings. Families that had already taken part in another family skills training programme in the past 24 months or where the caregiver lived separately from the child were excluded from the programme.

Allocation to the intervention or control group was done only after the first data collection, the only criteria used for allocation in either arm was convenience, i.e. availability of families (hence the allocation was non-biased). Participants in the intervention group were then told to attend the first programme session in the following week, whereas families in the control group were informed to attend the next data collection point 6 weeks later (2 weeks after the intervention group had completed the programme).

### Facilitator training & procedure

As a first step in Bangladesh, UNODC introduced the Family UNited programme to the government (Ministry of Secondary and Higher Education (MoE), Ministry of Primary and Mass Education, Ministry of Social Welfare and the Department of Narcotics Control). Focal points from the different Ministries along with civil society organizations (CSOs) were sensitized on the practical implementation of the pilot.

In June 2019 facilitators from Bangladesh (12 facilitators (3 male and 9 female)) and Indonesia (23 facilitators (7 male and 16 female), BNN prevention officers, practitioners in the prevention area and academia involved in the implementation study) were trained jointly on the Family UNited programme in Jakarta, Indonesia, by four international trainers/developers of the programme. All facilitators were then asked to implement the first pilot of Family UNited in their respective settings.

The MoE in Bangladesh identified the Government Laboratory High School in Dhaka as the first piloting site. In total, 48 families were recruited to participate in the programme. As this was the first time that Family UNited, a newly developed programme, was piloted with real families, the main aim at this stage was to guide the process evaluation of the programme (ease of applicability, content, length, and training qualifications) before moving to data collection and impact evaluation. The pre-pilot received appreciation from the Government as well as from the families who participated in the programme.

After that, a remote debriefing session was held by the lead developers with facilitators from Bangladesh in order to consolidate feedback received from the process evaluation. In October 2019, 15 facilitators (5 male and 10 female; 10 previously trained and 5 new facilitators) from Bangladesh and 24 facilitators (16 females and 8 males) from Indonesia were regrouped in a regional training in Jakarta, Indonesia, and re-trained on the revised materials to initiate the second phase of the impact evaluation of the programme. In addition, three research assistants from Bangladesh and six from Indonesia were trained on recruitment of participants, data collection and were acquainted with the data collection tools.

Based on the results of the national self-resilience mapping for 34 provinces in Indonesia [45], West Java Province was regarded below the average Adolescent Self-Resistance (Anti-Drug) Index in Indonesia. Therefore, West Java was chosen as the location for the implementation of the anti-drug family resilience model. BNN coordinated with BNN West Java Province, a research team from the Jakarta State University and the local Education Office to select and invite participants to the programm according to the selection criteria.

In the first phase of the Family UNited pilot in Indonesia, the implementation was carried out in four locations, namely Bandung City, Cianjur Regency, Cimahi City, and West Bandung Regency. The selection of the cities/regencies was based on the recommendations from the Education Office and the Narcotics Agency West Java. Furthermore, BNN then selected one Junior High School in each district/city. After the schools were selected, the principals chose 80 families according to the inclusion criteria, who were then informed and asked for their interest and willingness in participating in the Family UNited programme. According to this, 40 families were allocated to the intervention group, whereas those who only agreed to undertake the data collection but not the Family UNited programme, were allocated to the control group.

In Bangladesh, the MoE, Ministry of Home Affairs and Ministry of Social Welfare were involved in the implementation of Family UNited. Dhaka Ahsania Mission (DAM), as a private non-governmental organisation, was commissioned to conduct the pilot study. The MoE selected a school in Dhaka (Tejgaon High school) for the implementation. Families with children in that school were regarded as middle-class, with no major socioeconomic, cultural or ethnic differences between the families to be expected. The school was selected as it was large in size and rooms were available to conduct the Family UNited sessions smoothly. After selection of the school, DAM contacted the school authority and provided an overview of the FU programme, as well as the planned research. The school supported the selection of the families considering the inclusion criteria. Research assistants hung up banners in front of the main gate of the schools and inside the classrooms and an information sheet in Bengali containing information to caregivers of all children aged eight to 15 years was distributed in the school and caregivers were invited to an orientation meeting. School authorities arranged this meeting in the school auditorium, and the Family UNited research team provided an overview of the programme and the envisioned research to families. Overall, 40 families attended this orientation meeting, and 29 families agreed to attend the FU programme. Once families agreed to take part in the study, they attended a baseline measurement session in which written informed consent was obtained prior to data collection.

**Fig. 2:**
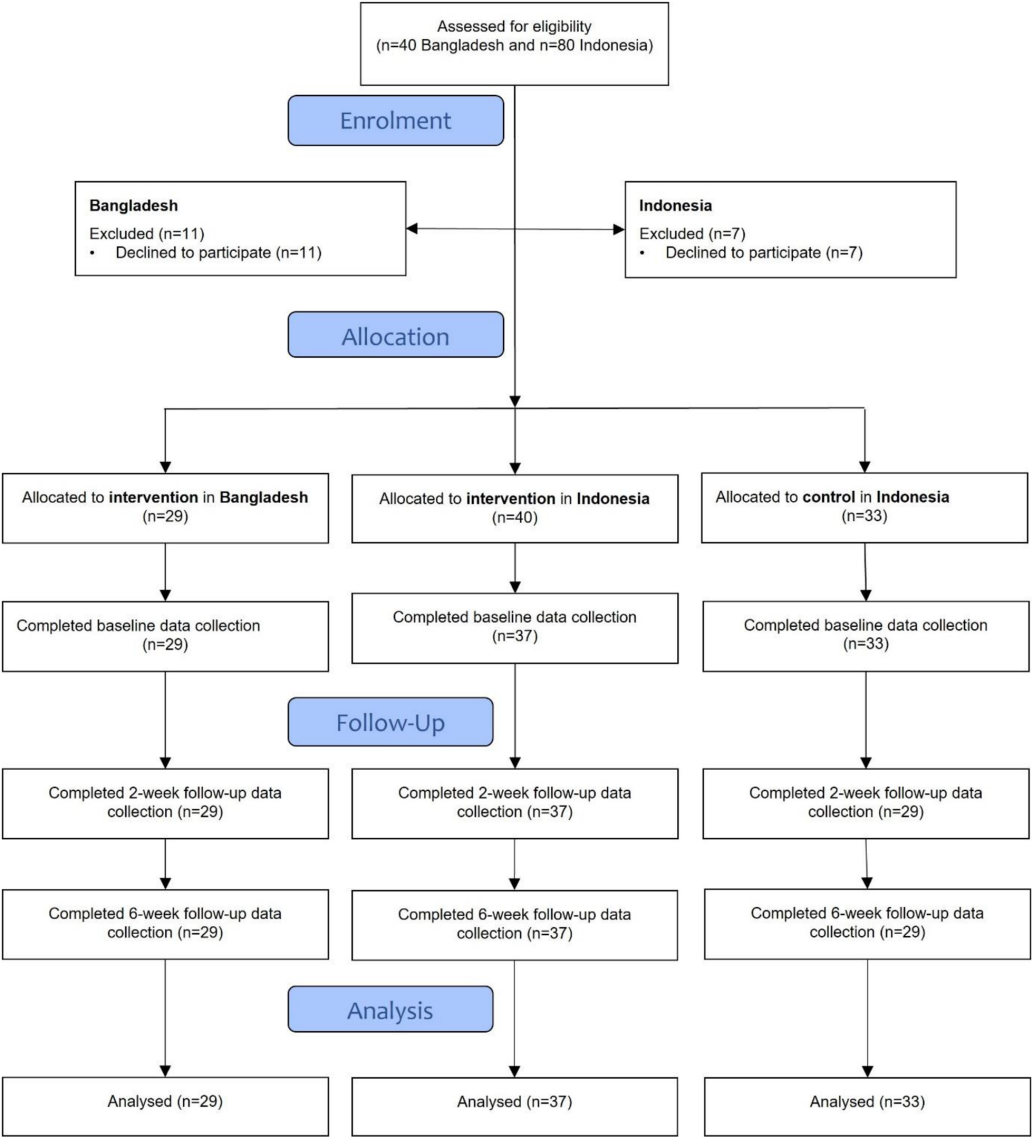
Modified CONSORT flow diagram

Overall, we enrolled families into the study in July 2019 in Indonesia and in November 2019 in Bangladesh, as shown in Fig. 2.

### Sample size

Based on our previous experience with the same measures assessing the effects of the Strong Families programme over time [10, 24, 25], a sample size of 30 was calculated for each group, keeping the power at 80% and the 2-sided confidence interval at 95% [46].

### Data collection

Caregivers provided data on emotional and behavioural difficulties of children and parental and family adjustment skills through self-administered questionnaires, whereas social-ecological resilience was self-reported through children in Indonesia.

One week before the start of the Family UNited programme (t1), a standardized Family Demographic Questionnaire (FDQ) was completed to collect baseline characteristics in Bangladesh. The FDQ had been previously used in other country contexts, such as Afghanistan [30], Uzbekistan, Zanzibar, Serbia, the Philippines, Iran [10] and the Dominican Republic, and only minor changes in some of the questions were made to reflect the Bangladeshi context. It was translated to Bangla by the DAM research team and a cultural working group and local advisory committee ensured and approved appropriateness. In Indonesia only the gender of the caregiver and child was recorded.

During this first data collection meeting (t1), the three outcome measures were also completed, and were further filled in at 2 weeks (t2) and 6 weeks (t3) after completion of the programme. These three measures, the Parenting and Family Adjustment Scales (PAFAS), the Strengths and Difficulties Questionnaire (SDQ) and the Child and Youth Resilience Measure (CYRM-R) were distributed as paper-based questionnaires. For the control group in Indonesia, the same measures were taken at the same timepoints, however no intervention was delivered in between. All families in the intervention and control group were unaware of their group allocation when filling in the questionnaires at t1.

The PAFAS questionnaire consists of 30-items, measuring parenting practices and family functioning, which are indicators for risk or protective factors for emotional or behavioural problems in children. It comprises two scales:(i) Parenting, measuring parenting practices and the quality of parent–child relationships within 4 subscales (“Parental Consistency” [Range: 0–15], “Coercive parenting” [0–15], “Positive encouragement” [0–9], “Parent–child-relationship” [0–15]) and (ii) Family Adjustment, measuring “parental adjustment” [0–15], “family relationships” [0–12] and “parental teamwork” [0–9] on the respective subscales. PAFAS was developed to assess changes in parenting skills and family relationships before and after individual or group parenting interventions and it has shown good internal consistencies and satisfactory construct and predictive validity in various country contexts [47–49]. In 2018, it has also been validated in 210 Indonesian parents with children aged 2–12 years old, with good to acceptable internal consistencies and satisfactory psychometric properties [50]. A cut off in points at the 66th percentile at baseline was assumed to separate participants with higher levels of difficulties for analyses purposes. The questionnaire was translated to Bangla by the DAM research team and was proof-read by the UNODC research team. The previously validated questionnaire in Bahasa was used in Indonesia.

The SDQ consists of 25 questions which are added into five different subscales indicating emotional symptoms, conduct problems, hyperactivity, peer problems and prosocial behaviors in children. The Total Difficulties score is calculated from four of the subscales excluding prosocial behaviours [51]. The SDQ has been used widely globally and is available in more than 40 different languages to examine children’s mental well-being. It has been validated in Indonesia in 2013 [52] and we used the common translations into Bangla and Bahasa [53] as well as the previously reported 4-banded categorization [54].

The CYRM-R is a self-report measure of social-ecological resilience consisting of 17 items adding up to the overall resilience score ranging from 17 to 85 points, with higher scores indicating higher levels of resilience [55]. The “overall resilience score” is the sum of the “caregiver resilience score” (ranging from 7 to 35 points) which relates to characteristics associated with the important relationships shared with the primary caregiver and the “personal resilience score” (10 to 50 points) which includes intra- and interpersonal items. We used the 5-point child version [56] with additional pictorial scales of glasses of water, as previously used in Syrian refugee and Jordanian host-community adolescents [57, 58]. CYRM-R has been tested for validity and reliability in 130 elementary school aged 10–13 years in Indonesia and has been recommended for research and practice in this context [59]. As shown in the logic model of the Family UNited programme (Fig. 1), with the CYRM-R we aimed to measure the short-term impacts such as “Reduced aggressive and hostile behaviors”, “Increased capacity to cope with stress” and “Improved mental health outcomes in children”. We separated children with low scores (≤ 33rd percentile) at baselines for additional analysis, as, to our knowledge, no clear cut-offs or thresholds have been recommended by the developers [55].

### Statistical analyses

All data was entered in Microsoft Excel and analysed using SPSS (version 26; IBM, Armonk, NY, USA). Plausibility checks were performed, and data completeness was assured prior to analyses. We did not impute data for the outcome variables of the three scales, as it was considered valid to ignore missing data [60]. Normality of data distribution on our multi-item Likert-type scales was assured through visual inspection of the histograms, Normal Q-Q plots and box plots and Kolmogorov–Smirnov tests. Continuous variables are presented as mean and standard deviation (SD) whereas categorical data were summarized as frequencies and proportions and compared using a chi-square test.

To compare scores at the different time points, we first tested a potential group-interaction effect through a two-way mixed ANOVA with within and between subjects’ factors. We further tested the effects of the respective outcome variable for families in the intervention and control group separately through a repeated measures ANOVA, accounting also for potential non-homogeneity of covariances, with post-hoc tests using Bonferroni corrections. In case Mauchly's Test of Sphericity indicated that the assumption of sphericity had been violated, a Huynh–Feldt correction was used. Homogeneity of Variances was tested through a Levene's test [61]. Results are reported separately for families living in Indonesia and Bangladesh. Participants with worse scores at baseline (regarded as families with more problems at baseline) were analysed separately for each of the subscales.

All data were analysed following the intention-to-treat approach. Statistical significance level was set at p-value lower than 0.05.

## Results

We enrolled families into the study and delivered the intervention in December 2019 in Bangladesh and between August and September 2019 in Indonesia, as shown in Fig. 2. The trial was ended after the intervention was completed after the 4 week period and all data were collected.

**Fig. 3:**
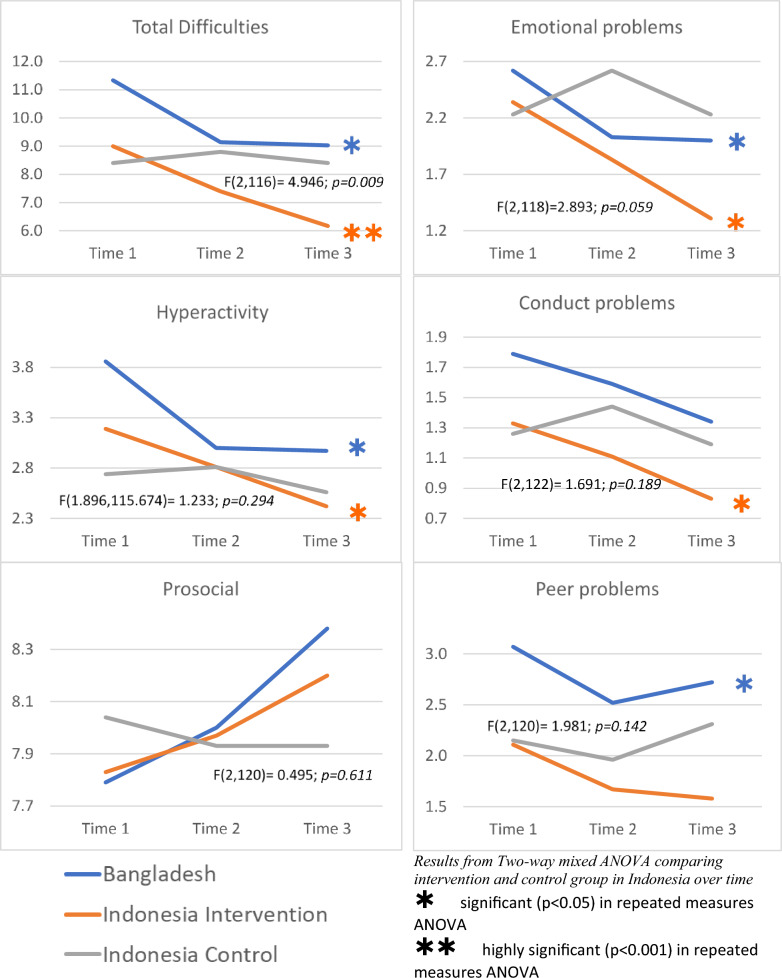
Overall SDQ results in Bangladesh and in the intervention and control group in Indonesia (higher scores indicating higher levels of difficulties on all subscales and the Total Difficulty Scale, except for the prosocial scale where higher scores indicate fewer difficulties)

### Description of missing data and loss to follow-up

Overall, 29 families in Bangladesh were enrolled in the study. In Indonesia, 37 completed the baseline data collection and were enrolled in the intervention group, and 33 in the control group. Follow-up was 100% in Bangladesh and in the intervention group in Indonesia, whereas it was 88% in the control group in Indonesia (Fig. 2).

Overall, there was very little missing data, with less than 5% of all individual questions missing at any timepoint (a maximum of 1.5% on the all PAFAS, 4.5% on the SDQ and 1.5% on the CYRM-R).

### Description of demographics

Families in the intervention and control group in Indonesia did not differ with respect to gender of the child taking part in the programme, however there were more fathers in the control group than the intervention group, as shown in Table 2.

**Table 2:** Demographic characteristics of study participants in Indonesia and Bangladesh

		Bangladesh n = 29	Indonesia (Intervention) n = 37	Indonesia (Control) n = 33	Comparison of intervention and control (Indonesia)	
		n (%)	n (%)	n (%)	p-value	Chi^2^
Gender of caregiver	Male	8 (28)	2 (5)	7 (22)	*0.043*	Χ^2^ = 4.104
	Female	21 (72)	35 (95)	25 (78)		
Gender of child in the programme	Male	16 (55)	21 (57)	15 (45)	*0.345*	χ^2^ = 0.892
	Female	13 (45)	16 (43)	18 (55)		

We collected demographic data from the eight fathers and 21 mothers in Bangladesh respectively. On average, caregivers were 40.3 years (± 5.73; range 29–55 years) old, with fathers being significantly older (45.6 vs 38.3 years). Overall, 93% of caregivers were married, 17% had a university degree, 10% a post-graduate degree, 7% trade or technical college qualification, 34% completed high school, 24% had some high school and 7% primary school or less. Twenty eight percent of caregivers were working full time, 3% part-time, 21% were not working but looking for a job, 10% were doing home-based paid work and 38% were not working. On average, our recruited caregivers had 2.34 ± 1.14 children (Range: 1–6 children) to care for, and the average age of children taking part in the programme was 13.03 ± 1.40 years (Range: 11–15 years). All caregivers reported Bangladesh as their country of origin and one caregiver had experienced war or armed conflict in his past.

### Parenting skills

#### Overall parenting and family adjustment skills results

In Bangladesh, we found reductions in mean scores on all subscales, however only being statistically significantly reduced in the positive encouragement and parent–child-relationship subscale. In Indonesia, there was a statistically significant interaction between the groups and time on the coercive parenting, positive encouragement, parent–child relationship and family relationships subscales. Overall, there was no effect over time in the control group on any of the subscales, whereas significant reductions in scores were found on six of the seven subscales in the intervention group in Indonesia, as shown in Table 3.

**Table 3:** Mean PAFAS scores over time by country and intervention or control group

PAFAS		Pre-test mean (SD)	Post-test mean (SD)	Follow-up mean (SD)	Two-way mixed ANOVA F(df_time_, df_error_); p-value	Repeated measures ANOVA F(df_time_, df_error_); p-value	Post-hoc tests
Parenting							
Parental consistency [0–15]							
Bangladesh	Intervention (n = 29)	8.83 (1.93)	8.21 (1.84)	8.21 (1.63)	n/a		
Indonesia	Intervention (n = 37)	6.57 (1.77)	5.70 (1.94)	5.62 (2.03)	F(2,128) = 0.470; *p* = *0.626*	F(2,72) = 4.334; *p* = *0.017*	
	Control (n = 29)	7.00 (1.10)	6.48 (1.64)	6.48 (1.55)			
Coercive parenting [0–15]							
Bangladesh	Intervention (n = 29)	3.97 (1.99)	3.90 (2.80)	3.34 (2.99)	n/a		
Indonesia	Intervention (n = 36)	5.25 (2.50)	4.00 (2.11)	3.56 (2.32)	F(2,122) = .628; *p* = *0.029*	F(2,70) = 12.125; *p* < *0.001*	✱ ■
	Control (n = 27)	4.93 (2.24)	4.85 (2.13)	4.59 (1.95)			
Positive encouragement [0–9]							
Bangladesh	Intervention (n = 29)	2.10 (1.92)	1.17 (1.58)	1.07 (1.44)	n/a	F(1.456,40.754) = 5.618; *p* = *0.013*	■
Indonesia	Intervention (n = 37)	2.51 (2.01)	1.51 (1.26)	1.59 (1.30)	F(2,128) = 5.171; *p* = *0.007*	F(1.701,61.230) = 6.858; *p* = *0.003*	✱ ■
	Control (n = 29)	2.62 (1.97)	2.79 (1.68)	2.93 (1.53)			
Parent–child relationship [0–15]							
Bangladesh	Intervention (n = 29)	2.45 (3.11)	1.76 (2.36)	1.34 (2.01)	n/a	F(1.526,42.742) = 3.923; *p* = *0.038*	
Indonesia	Intervention (n = 37)	1.65 (2.67)	0.92 (1.34)	0.92 (1.30)	F(1.360,87.010) = 3.706; *p* = *0.045*		
	Control (n = 29)	1.62 (1.80)	2.07 (1.65)	2.17 (1.93)			
Family adjustment							
Parental adjustment [0–15]							
Bangladesh	Intervention (n = 29)	4.48 (3.00)	4.48 (2.59)	3.72 (2.09)	n/a		
Indonesia	Intervention (n = 37)	4.00 (2.36)	3.41 (2.03)	3.03 (2.29)	F(1.825,115.001) = 2.190; *p* = *0.121*	F(1.709,61.528) = 5.422; *p* = *0.010*	■
	Control (n = 28)	3.50 (2.56)	3.43 (2.67)	3.54 (2.15)			
Family relationships [0–12]							
Bangladesh	Intervention (n = 29)	1.93 (1.83)	1.41 (1.74)	1.38 (1.50)	n/a		
Indonesia	Intervention (n = 37)	1.30 (1.81)	1.03 (1.48)	0.97 (1.55)	F1.906,120.085) = 3.917; *p* = *0.024*		
	Control (n = 28)	1.00 (1.19)	1.54 (1.43)	1.39 (1.45)			
Parental teamwork [0–9]							
Bangladesh	Intervention (n = 28)	1,82 (1,61)	1,68 (1,39)	1,50 (1,29)	n/a		
Indonesia	Intervention (n = 34)	2,35 (2,31)	0,94 (1,37)	1,32 (1,55)	n/a	F(1.726,56.964) = 8.362; *p* = *0.001*	
	Control (n = 0)						✱

Statistically significant (p < 0.05), higher PAFAS scores indicating lower levels of parenting and family adjustment skills; results for repeated measure ANOVAs and post-hoc tests only shown if significant; SD: standard deviation; ✱ significant difference between t1 and t2, ■ significant difference between t1 and t3

#### Parenting and family adjustment skills results in caregivers above the 66th percentile

As shown in Table 3, mean PAFAS scores were different between families in Bangladesh and Indonesia in many of the subscales. Therefore, caregivers above the 66th percentile were analysed separately for each country and cut-offs and results are presented in Table 4. Again, families improved on six of the seven subscales in either country in the intervention group, whereas there was no change in the control group, apart from the coercive parenting subscale.

**Table 4:** Total difficulty scores in boys and girls in Bangladesh and Indonesia over time.

PAFAS in caregivers above the 66th percentile at pre-test		Pre-test mean (SD)	Post-test mean (SD)	Follow-up mean (SD)	Two-way mixed ANOVA F(df_time_, df_error_); p-value	Repeated measures ANOVA F(df_time_, df_error_); p-value	Post-hoc tests
Parenting							
Parental Consistency							
Bangladesh	Intervention (n = 10)	10.90 (1.20)	9.20 (1.40)	8.70 (1.64)	n/a	F(2,18) = 9.184; *p* = *0.002*	✱ ■
Indonesia	Intervention (n = 13)	8.31 (0.63)	5.69 (1.84)	6.00 (2.58)	F(2,38) = 1.923; *p* = *0.160*	F(2,24) = 10.140; *p* = *0.001*	✱ ■
	Control (n = 8)	8.50 (0.54)	7.62 (0.74)	7.38 (1.69)			
Coercive parenting							
Bangladesh	Intervention (n = 10)	6.20 (1.14)	3.90 (1.52)	4.10 (2.47)	n/a	F(2,18) = 6.513; *p* = *0.007*	✱
Indonesia	Intervention (n = 20)	7.10 (1.25)	4.90 (2.08)	4.30 (2.56)	F(2,56) = 1.510; *p* = *0.230*	F(2,38) = 23.462; *p* < *0.001*	✱ ■
	Control (n = 10)	7.20 (1.40)	6.30 (2.00)	5.20 (1.69)		F(2,18) = 5.026; *p* = *0.018*	■
Positive encouragement							
Bangladesh	Intervention (n = 12)	4.00 (1.28)	1.75 (2.01)	1.58 (1.68)	n/a	F(2,22) = 8.446; *p* = *0.002*	✱ ■
Indonesia	Intervention (n = 10)	5.00 (1.70)	2.20 (1.62)	2.00 (1.41)	F(2,36) = 4.490; *p* = *0.018*	F(2,18) = 11.073; *p* = *0.001*	✱ ■
	Control (n = 10)	4.80 (1.03)	3.60 (1.08)	4.20 (0.79)		F(2,18) = 4.585; *p* = *0.025*	
Parent–child relationship							
Bangladesh	Intervention (n = 11)	5.55 (3.01)	3.55 (2.73)	2.82 (2.60)	n/a	F(2,20) = 5.343; *p* = *0.014*	
Indonesia	Intervention (n = 14)	3.79 (3.38)	1.14 (1.41)	1.29 (1.14)	F(1.302,33.852) = 4.957; *p* = *0.024*	F(1.261,16.396) = 5.907; *p* = *0.021*	
	Control (n = 14)	3.14 (1.41)	3.07 (1.27)	3.29 (1.68)			
Family adjustment							
Parental adjustment							
Bangladesh	Intervention (n = 12)	7.42 (1.78)	5.25 (2.56)	4.50 (2.20)	n/a	F(2,22) = 11.888; *p* < *0.001*	✱ ■
Indonesia	Intervention (n = 13)	6.54 (1.56)	4.46 (1.61)	4.38 (2.26)	F(1.734,36.421) = 0.380; *p* = *0.657*	F(2,24) = 14.628; *p* < *0.001*	✱ ■
	Control (n = 10)	6.30 (1.64)	4.90 (2.81)	4.40 (2.01)			
Family relationships							
Bangladesh	Intervention (n = 10)	4.00 (1.25)	2.40 (2.01)	2.30 (1.77)	n/a	F(2,18) = 6.882; *p* = *0.006*	✱ ■
Indonesia	Intervention (n = 7)	4.43 (2.07)	2.43 (2.64)	2.29 (2.75)	F(1.594,20.718) = 4.008; *p* = *0.042*	F(1.029,6.176) = 9.108; *p* = *0.022*	
	Control (n = 8)	2.63 (0.74)	2.38 (1.60)	2.38 (1.51)			
Parental teamwork							
Bangladesh	Intervention (n = 7)	4.00 (1.16)	2.86 (1.35)	1.71 (1.11)	n/a	F(2,12) = 8.862; *p* = *0.004*	■
Indonesia	Intervention (n = 13)	4.92 (1.55)	1.54 (1.71)	1.77 (1.88)	n/a	F(2,24) = 22.452; *p* < *0.001*	
	Control (n = 0)						✱ ■

Statistically significant (p < 0.05), Results for repeated measure ANOVAs and post-hoc tests only shown if significant; SD: standard deviation; ✱ significant difference between t1 and t2, ■ significant difference between t1 and t3

### Child mental health

#### Overall SDQ results in Bangladesh and in the intervention and control group in Indonesia

Overall, there were (highly) significant changes in scores in the intervention group on the “total difficulty scale “ in both countries, whereas there was no effect in the control group in Indonesia. There were significant improvements in scores in three of the four subscales in Bangladesh and in the intervention group in Indonesia, which could not be found in any of the subscales in the control group, as shown in Fig. 3

**Fig. 4:**
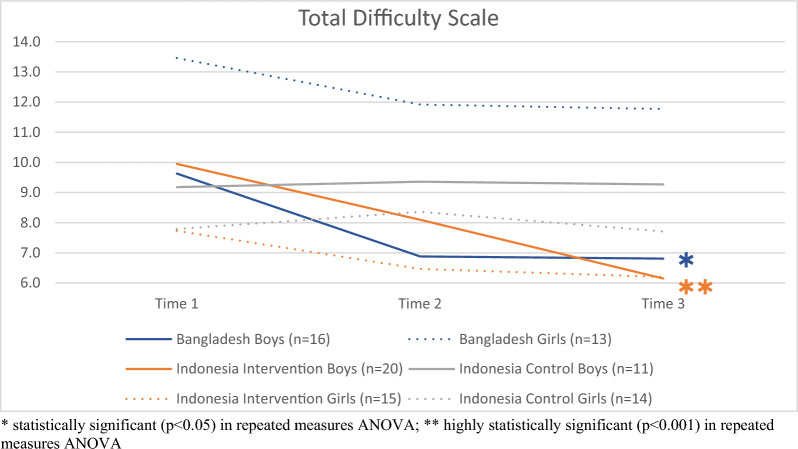
Total difficulty scores in boys and girls in Bangladesh and Indonesia over time

#### Mental health results in children with high and very high total scores at baseline

Despite the very small number of children with high or very high total difficulty scores at baseline, we saw a declining trend over time in Bangladesh and in the intervention group in Indonesia, as shown in Table 5.

**Table 5:** Total difficulty scores in children with 17 or more points at pre-test

SDQ		Pre-test Mean (SD)	Post-test Mean (SD)	Follow-up Mean (SD)	Two-way mixed ANOVA F(df_time_, df_error_); p-value	Repeated measures ANOVA F(df_time_, df_error_); p-value	Post-hoc tests
Total difficulty scores in children with 17 or more points at pre-test (High and very high)							
Bangladesh	Intervention (n = 4)	21.5 (5.07)	16.5 (4.04)	16.3 (3.59)	n/a		
Indonesia	Intervention (n = 3)	19.0 (3.46)	13.7 (1.53)	14.3 (2.52)	F(2,4) = 0.776; p = 0.519		
	Control (n = 1)	18.0	18.0	18.0			

#### Child mental health results by gender

Girls in Bangladesh started off at the highest level of all children with an average of 13.5 points on the total difficulty scale at baseline. Although not statistically significant, they improved in scores after the programme. Boys in Bangladesh and boys in the intervention group in Indonesia however improved (highly) significantly in scores after the intervention, an effect that could not be found in the control group in either gender, as shown in Fig. 4. Likewise, these effects could be found in almost all subscales in boys in the intervention group in Indonesia and in Bangladesh, but not in girls and neither in the control group (data not shown).

### Child resilience

#### Overall child resilience scores in Bangladesh and in the intervention and control group in Indonesia

All children in both countries were already in the “high resilience” category at baseline when cautiously applying the Canadian thresholds [55]. Children in the intervention group in Indonesia improved significantly on both the caregiver resilience and the personal resilience subscale, as well as on the overall resilience scale over time, an effect that could not be seen in the control group. Children in Bangladesh improved in scores of the overall resilience scale, however only improved statistically significantly on the personal resilience subscale, as shown in Table 6.

**Table 6:** Mean CYRM-R scores over time by country and intervention or control group

CYRM-R		Pre-test mean (SD)	Post-test mean (SD)	Follow-up mean (SD)	Two-way mixed ANOVA F(df_time_, df_error_); p-value	Repeated measures ANOVA F(df_time_, df_error_); p-value	Post-hoc tests
Personal resilience subscale [10–50]							
Bangladesh	Intervention (n = 29)	41.14 (5.62)	42.59 (5.88)	43.21 (4.95)	n/a	F(2,56) = 4.352; *p* = *0.018*	■
Indonesia	Intervention (n = 37)	39.57 (4.00)	42.14 (4.47)	41.70 (4.92)	F(2,126) = 3.478; *p* = *0.034*	F(1.769,63.668) = 8.798; *p* = *0.001*	✱ ■
	Control (n = 28)	40.29 (4.62)	40.29 (5.58)	40.82 (5.29)			
Caregiver resilience subscale [7–35]							
Bangladesh	Intervention (n = 29)	31.17 (4.20)	31.31 (4.02)	31.03 (3.81)	n/a		
Indonesia	Intervention (n = 37)	31.27 (2.93)	32.19 (3.04)	32.05 (3.46)	F(2,128) = 0.464; *p* = *0.630*	F(1.804,64.955) = 3.774; *p* = *0.032*	
	Control (n = 29)	31.21 (2.92)	31.59 (3.31)	31.59 (3.46)			
Overall resilience scale [17–85]							
Bangladesh	Intervention (n = 29)	72.31 (9.44)	73.90 (9.29)	74.24 (7.69)	n/a		
Indonesia	Intervention (n = 37)	70.84 (6.00)	74.32 (6.94)	73.76 (7.70)	F(2,126) = 2.493; *p* = *0.087*	F(1.642,59.119) = 8.635; *p* = *0.001*	
	Control (n = 28)	71.43 (6.60)	71.86 (7.96)	72.32 (7.84)			✱ ■

Statistically significant (p < 0.05); higher scores indicating a higher levels of resilience; Results for repeated measure ANOVAs and post-hoc tests only shown if significant; SD: standard deviation; ✱ significant difference between t1 and t2, ■ significant difference between t1 and t3

### Resilience results in children below the 33rd percentile

In children who had CYRM-R scores below the 33rd percentile at baseline, we found a (highly) significant improvement over time on the “personal resilience subscale” and the overall resilience scale in the intervention group in Indonesia and in Bangladesh, which was not found in the control group, as shown in Table 7.

**Table 7:** Mean CYRM-R scores over time in children below the 33rd percentile at pre-test

CYRM-R in children below the 33rd percentile at pre-test		Pre-test mean (SD)	Post-test mean (SD)	Follow-up mean (SD)	Two-way mixed ANOVA F(df_time_, df_error_); p-value	Repeated measures ANOVA F(df_time_, df_error_); p-value	Post-hoc tests
Personal resilience subscale [10–50]							
Bangladesh	Intervention (n = 9)	34.33 (3.35)	38.56 (4.77)	39.89 (3.66)	n/a	F(2,16) = 9.418; *p* = *0.002*	✱ ■
Indonesia	Intervention (n = 21)	34.62 (3.23)	39.14 (4.46)	39.19 (4.37)	F(2,54) = 3.137; *p* = *0.051*	F(2,40) = 19.404; *p* < *0.001*	✱ ■
	Control (n = 8)	34.75 (3.45)	35.50 (4.87)	35.75 (5.39)			
Caregiver resilience subscale [7–35]							
Bangladesh	Intervention (n = 8)	26.00 (4.41)	28.50 (3.74)	28.13 (3.09)	n/a		
Indonesia	Intervention (n = 15)	26.53 (4.14)	28.87 (4.47)	28.07 (4.17)	F(2,46) = 0.586; *p* = *0.560*		
	Control (n = 10)	27.80 (1.32)	28.50 (3.47)	28.80 (2.97)			
Overall resilience scale [17–85]							
Bangladesh	Intervention (n = 9)	61.00 (7.40)	68.56 (7.40)	68.22 (5.29)	n/a	F(2,16) = 9.011; *p* = *0.002*	✱ ■
Indonesia	Intervention (n = 18)	62.11 (7.21)	68.94 (7.60)	69.17 (7.69)	F(2,50) = 1.915; *p* = *0.158*	F(2,34) = 19.816; *p* < *0.001*	✱ ■
	Control (n = 9)	64.00 (3.50)	66.56 (7.80)	67.00 (8.73)			

Statistically significant (p < 0.05), Results for repeated measure ANOVAs and post-hoc tests only shown if significant; SD: standard deviation; ✱ significant difference between t1 and t2, ■ significant difference between t1 and t3

### Child resilience results by gender

**Fig. 5:**
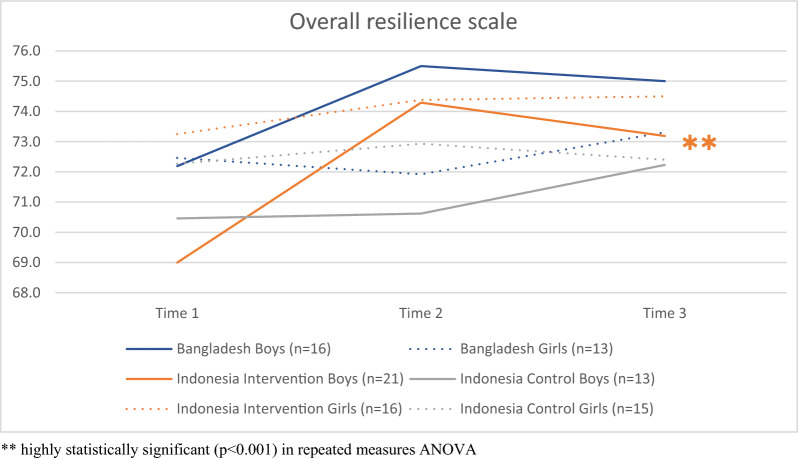
Overall resilience scale in boys and girls in Bangladesh and Indonesia over time

There was a highly significant improvement in scores in boys in the intervention group in Indonesia over time, which was not found in the control group (Fig. 5). These changes in boys were due to improvements in both, the personal and caregiver resilience subscale over time. In addition, boys in Bangladesh also improved significantly on the personal resilience subscale.

## Discussion

This multisite pilot of Family UNited aimed to assess the feasibility and effectiveness of the Family UNited package in two different pilot sites. The overall pilot, across sites, reflected a significant improvement in scores amongst those benefiting from the package on all three measurements scales. This was noted in Bangladesh where only an intervention arm was planned as well as in Indonesia where a comparison group was added to contrast the changes noticed in the intervention group. The fact that these changes were noted despite the differences in country context, and the difference in scores at baseline, is reassuring. What was more valuable, was that these beneficial changes in scores were more prominent in families with higher problems at start point, as previously found in other settings [10, 24, 25].

Although we found promising changes in girls on the SDQ and CYRM-R measurements, it seems that boys seem to have particularly benefitted from the outcome of this package, hence urging the need to offer such programmes irrespective of gender or “difficulty” of the child. In previous research with family skills interventions in Low and Middle Income countries, interventions have had similar positive significant effects irrespective of gender [10]

Despite the differences in country contexts between Bangladesh and Indonesia, the fact that families in both countries benefitted from the intervention adds value to the effectiveness component of the research. Despite the absence of a control group in Bangladesh, most data parallel the ones from the intervention group in Indonesia. Nevertheless, future research including a control group in Bangladesh would further benefit the results.

Comparing SDQ and PAFAS scores at baseline in Indonesia and Bangladesh to families we recruited for the “Strong Families” programme in Afghanistan [24], in refugee reception centres in Serbia [25] and in Iran [10], we can say that our current caregivers in this study started off with much lower scores on the coercive parenting subscale (3.97 in Bangladesh, 5.25 in the intervention group in Indonesia and 4.93 in the control group, compared to 8.49 in Afghanistan, 8.32 in Serbia and 7.39 and 6.87 in the Iranian intervention and control groups respectively). This was expected given the universal application of Family UNited vs. the selective application of Strong Families. Nevertheless, despite the lower scores at baseline in this study, we saw highly significant improvements in the intervention group in Indonesia, accounting for the beneficial effects even in such universal context. A similar phenomenon was found on the parental adjustment and the family relationships subscales of the PAFAS, as well as the Total SDQ, emotional problem and conduct problem scales of the SDQ.

As this was the first time, Family UNited was actually piloted, and given the fact that families and facilitators were new to this programme, the results of our effectiveness evaluation are reassuring in the sense that the programme moved the needles across scales in the favourable direction, irrespective of the setting, and despite early and brief experience of the trained facilitators with the content. This makes Family UNited particularly valuable for resource-limited settings, where facilitator time and training might be hampered. Facilitators did not require any formal education or training; hence we can advocate for scale up with motivated facilitators who have easy access to families. For sustainability purposes, integration of family skills programmes into routine services to families would be ideal and investment in facilitator training seems minimal compared to the long-term benefits to their portfolio they can offer to families.

Family skills interventions in general are recommended as a primary prevention measure, providing to be far more beneficial, and thus costs effective, than treatment of a number of short and long term challenges youth and adolescents may experience, from mental health challenges to substance use disorders and partaking in risky behaviours [62, 63]. The value of such family skills interventions was further corroborated by the guidelines on parenting interventions to prevent maltreatment and enhance parent–child relationships with children aged 0–17 years as recently launched the World Health Organization [64].

Although drivers for violent extremism are multi-factorial, [65] we aimed to measure resilience, as protective factor against its development. The indicators used, per the CYRM-R, were also reassuring and added further value to the Family UNited effectiveness, despite brevity of intervention. We regarded family as a key institution that can strengthen resilience during the developmental period and violence prevention was integrated into the logic model of the Family UNited programme, like other risky behaviours [24].

Recognizing the role that caregivers play as a key tool for improving outcomes and mitigating children’s exposure to low resource induced risk and harm is very important. Similarly, these results also induce parents living in challenged settings to further engage in their social role with the family and improve care, monitoring, communication, reciprocal support, particularly given the difficult living conditions they are living in.

## Limitations

Despite all reassuring results, our study has a few limitations. Firstly, the samples were opportunistic which means the beneficiaries are not necessarily representatives of either the Bangladesh or Indonesian families. Nevertheless, for the purpose of the research questions sought, the intent was to reflect cross-cultural effectiveness which to a large extend can be ensured from the fact that the nature of the families in each sample was different. Moreover, we unfortunately did not collect more demographic data in Indonesia and hence cannot compare them to caregivers in our sample in Bangladesh. Caregivers from Bangladesh seemed quite well educated, with 27% of caregivers in our sample having a university or even postgraduate degree. According to a Bangladesh government report from 2018 though, the literacy rate was as high as 74.4% in adults in Dhaka though, with males having a higher rate of 77.2 compared to women with 71.6% [78]. The school selected for our intervention was known to host middle-class families in Dhaka.

Secondly, despite including a comparison group in Indonesia, this was not a Randomized Control Trial per se. Still, for the purpose of answering this research objective, the presence of a comparison group in Indonesia to complement the intervention only results in Bangladesh, added insight on the effectiveness of Family UNited. However, future research will need to ensure proper matching through a Randomized Clinical Trial protocol and methodology in all further multi-site implementation research to add value on the impact of Family UNited on beneficiary families. This would increase likelihood of transferability to other global regions,

Although our programme and invitation for participation was universally targeted, our sample consisted of more mothers. The lack of participation of fathers in parenting research and implementation is a common limitation in the parenting literature. This is even more evident in settings of humanitarian or underserved contexts. We, therefore, cannot generalise the findings to the male caregiver population. However, future research should shed light onto the role of fathers/male caregivers in family skills programmes and investigate the potential of including both caregivers in such programmes.

Overall, and accounting for the above limitations, with these encouraging results, UNODC intends to further extends such pilots to further sites within the countries of concern as well as in other countries hoping to further replicate and corroborate findings.

## Conclusions

With our pilot study we showed that the implementation of this new, brief, open sourced and universal family skills programme designed for Low and Middle Income Countries was feasible and showed promising effects in two countries in Southeast Asia. Transferability to other global regions and further research gathering data on the long-term effects and enhanced comparison through a randomised controlled trial needs to be undertaken to recommend scalability at a larger global level. The Family UNited brevity, while encouraging easier acceptance in Low and Middle Income Countries where the infrastructure is relatively limited needs to be further explored in its potential for moving to scale. The biopsychosocial vulnerability, resiliency, and social learning theory models that guide its content indicate that such short-term changes, at the family level, are good indicators, in support of longer-term impacts on child development that requires further research, particularly on the elements of reduction of violence, substance use, and mental health outcomes Nevertheless, the current findings, including replicability of findings from other countries, already suggest a strong advocacy message for stakeholders implicated in child mental health, resilience, and healthy and safe development, especially those living in particularly underprivileged circumstances, to consider the Family UNited programme as a package of support.

## Data Availability

The datasets generated and analysed during the current study are available in the Mendeley repository, https://data.mendeley.com/datasets/krxbjm89zr/1

## Abbreviations

ANOVA: Analysis of variance
BNN: Indonesian National Narcotics Board
CSOs: Civil society organizations
CYRM-R: Child and youth resilience measure
DAM: Dhaka Ahsania Mission
FDQ: Family demographic questionnaire
FU: Family UNited
MICs: Middle-income countries
MoE: Ministry of secondary and higher education
PAFAS: Parenting and family adjustment scales
RCT: Randomized controlled trial
SD: Standard deviation
SDQ: Strengths and difficulties questionnaire
UNODC: United Nations Office on Drugs and Crime
WHO: World Health Organisation

## References

[1] Byrne ML (2017). Self-reported parenting style is associated with children's inflammation and immune activation. J Fam Psychol.

[2] Ryan R, O'Farrelly C, Ramchandani P (2017). Parenting and child mental health. London J Prim Care.

[3] Stormshak EA (2000). Parenting practices and child disruptive behavior problems in early elementary school psychol conduct problems prevention research group. J Clin Child.

[4] Parsons CE (2012). Postnatal depression and its effects on child development: a review of evidence from low- and middle-income countries. Br Med Bull.

[5] Yue A (2017). China’s invisible crisis: cognitive delays among rural toddlers and the absence of modern parenting. China J.

[6] Rutter M (1985). Resilience in the face of adversity protective factors and resistance to psychiatric disorder. Br J Psychiatry.

[7] Skogstad, E. A preliminary evaluation on the effectiveness of a universal school-based mindfulness intervention to enhance resilience in adolescents. 2017. Unitec: New Zealand. p. 84.

[8] Masten AS (2001). Ordinary magic resilience processes in development. Am Psychol.

[9] Höltge J (2021). A cross-country network analysis of adolescent resilience. J Adolesc Health.

[10] Haar K (2021). Impact of a brief family skills training programme (“strong families”) on parenting skills, child psychosocial functioning, and resilience in iran: a multisite controlled trial. Int J Environ Res Public Health.

[11] Weine S (2012). Building resilience to violent extremism in Muslim diaspora communities in the United States. Dynam Asymmetr Confl.

[12] Pedersen GA (2019). A Systematic review of the evidence for family and parenting interventions in low- and middle-income countries: child and youth mental health outcomes. J Child Fam Stud.

[13] World Bank (2016). World development report 2016 digital dividends.

[14] Knerr W, Gardner F, Cluver L (2013). Improving positive parenting skills and reducing harsh and abusive parenting in low- and middle-income countries: a systematic review. Prev Sci.

[15] WHO (2010). Violence prevention: the evidence.

[16] Biglan A (2012). The critical role of nurturing environments for promoting human well-being. Am Psychol.

[17] Barry M (2001). Promoting positive mental health: theoretical frameworks for practice. Int J Ment Health Promot.

[18] Maalouf W, Campello G (2014). The influence of family skills programmes on violence indicators: experience from a multi-site project of the United Nations office on drugs and crime in low and middle income countries. Aggress Violent Beh.

[19] Murphy K (2017). Raising children in conflict: an integrative model of parenting in war. Peace Confl J Peace Psychol.

[20] El-Khani A (2022). Bridging the gap between the pressing need for family skills programmes in Humanitarian Settings and Implementation. Int J Environ Res Public Health.

[21] Ponguta LA (2020). Effects of the mother-child education program on parenting stress and disciplinary practices among refugee and other marginalized communities in lebanon: a pilot randomized controlled trial. J Am Acad Child Adolesc Psychiatry.

[22] Miller KE (2020). Supporting Syrian families displaced by armed conflict: a pilot randomized controlled trial of the caregiver support intervention. Child Abuse Negl.

[23] Sim AL (2021). Acceptability and preliminary outcomes of a parenting intervention for Syrian refugees. Res Soc Work Pract.

[24] Haar K (2020). Strong families: a new family skills training programme for challenged and humanitarian settings: a single-arm intervention tested in Afghanistan. BMC Public Health.

[25] El-Khani A (2021). Assessing the feasibility of providing a family skills intervention, "strong families", for refugee families residing in reception centers in Serbia. Int J Environ Res Public Health..

[26] UNODC. Family UNited: Universal family skills programme for prevention of negative social outcomes in low—and middle-income countries. 2020. https://www.unodc.org/res/listen-first/parenting-under-covid-19_html/Family-UNited-leaflet-20200218.pdf.

[27] Duckworth AL (2007). Grit: Perseverance and passion for long-term goals. J Pers Soc Psychol.

[28] Silverman IW (2003). Gender differences in delay of gratification: a meta-analysis. Sex Roles.

[29] Kumpfer, K.L., E.P. Trunnell, and H. Whiteside. 1990. The Biopsychosocial Model. RC Engs (eds). Application to the Addictions Field in Controversies in the Addiction Field. Kendal-Hunt. Dubuque

[30] Richardson GE (1990). The resiliency model. Health Educ.

[31] Garmezy N (1991). Resilience in children's adaptation to negative life events and stressed environments. Pediatr Ann.

[32] Betancourt TS (2020). The intergenerational impact of war on mental health and psychosocial wellbeing: lessons from the longitudinal study of war-affected youth in Sierra Leone. Confl Heal.

[33] Bandura A, Walters RH (1977). Social learning theory.

[34] O'Connor TG (2013). Social learning theory parenting intervention promotes attachment-based caregiving in young children: randomized clinical trial. J Clin Child Adolesc Psychol.

[35] Scott SF (2015). Gardner parenting programs, in Rutter's child and adolescent psychiatry.

[36] The World Bank Group. Data, Bangladesh. 2019 5 2019. https://data.worldbank.org/country/bangladesh

[37] World Health Organisation (2019). Minister of health releases first findings of national mental health survey.

[38] Firoz AHM (2006). Prevalence, medical care, awareness and attitude towards mental illness in Bangladesh. Bangladesh Journal of Psychiatry.

[39] The World Bank in Indonesia. Country Overview. Oct 29. 2021. https://www.worldbank.org/en/country/indonesia/overview#1.

[40] UNODC, Country Programme of Indonesia 2017—2020. Making Indonesia safer from crime drugs and terrorism". 2017, United Nations Office on Drugs and Crime.

[41] KEMENTERIAN KESEHATAN REPUBLIK INDONESIA (Ministry of Health Republic of Indonesia). Pusat Data dan Informasi. 2019. https://pusdatin.kemkes.go.id/folder/view/01/structure-publikasi-data-pusat-data-dan-informasi.html.

[42] Saputra F, Yunibhand J, Sukratul S (2017). Relationship between personal, maternal, and familial factors with mental health problems in school-aged children in Aceh province. Indonesia Asian J Psychiatr.

[43] UNODC/WHO, International Standards on Drug Use Prevention, in Second updated edition (2018). United Nations office on drugs and crime.

[44] MIKTA. Prevention of narcotic drug abuse in MIKTA Countries. MIKTA side event in the margins of the 60th commission on narcotic drugs 2017. http://mikta.org/project/mikta-side-event-on-prevention-of-narcotic-drug-abuse-in-mikta-countries-17-march-2017-vienna/?ckattempt=1.

[45] National Narcotics Board (BNN), Research report on self resilience mapping (anti) drugs 2018.

[46] Dhand, N. and M. Khatkar. Statulator. An online statistical calculator. Sample size calculator for comparing two paired means. 2014. http://statulator.com/SampleSize/ss2PM.html#.

[47] Sanders MR (2014). Parenting and family adjustment Scales (PAFAS): validation of a brief parent-report measure for use in assessment of parenting skills and family relationships. Child Psychiatry Hum Dev.

[48] Mejia A (2015). Measuring parenting practices and family functioning with brief and simple instruments: validation of the Spanish version of the PAFAS. Child Psychiatry Hum Dev.

[49] Guo M, Morawska A, Filus A (2017). Validation of the parenting and family adjustment scales to measure parenting skills and family adjustment in Chinese parents. Meas Eval Couns Dev.

[50] Sumargi A (2017). The parenting and family adjustment scales (PAFAS): an Indonesian validation study. J Child Fam Stud.

[51] Goodman R (1997). The strengths and difficulties questionnaire: a research note. J Child Psychol Psychiat.

[52] Oktaviana M (2014). Wimbarti, *Validasi Klinik Strenghts and Difficulties Questionnaire (SDQ) sebagai Instrumen Skrining Gangguan Tingkah Laku*. Jurnal Psikologi..

[53] SDQInfo. Downloadable SDQs and related items. 2015 14/08/2020]. https://www.sdqinfo.org/py/sdqinfo/b0.py.

[54] EHCAP. Scoring the strengths & difficulties questionnaire for age 4–17. 2014 12/03/2019]. https://www.ehcap.co.uk/content/sites/ehcap/uploads/NewsDocuments/236/SDQEnglishUK4-17scoring-1.PDF.

[55] Resilience Research Centre CYRM and ARM user manual (2018). Resilience Research Centre.

[56] Jefferies P, McGarrigle L, Ungar M (2019). The CYRM-R: a rasch-validated revision of the child and youth resilience measure. J Evid Based Soc Work.

[57] Panter-Brick C (2018). Resilience in context: a brief and culturally grounded measure for syrian refugee and jordanian host-community adolescents. Child Dev.

[58] Wener RW (2016). Evaluating resilience in Syrian and Jordanian youth 2016.

[59] Borualogo IS, Jefferies P (2019). Adapting the child and youth resilience measure-revised for indonesian contexts. J Educ Health Commun Psychol.

[60] Jakobsen JC (2017). When and how should multiple imputation be used for handling missing data in randomised clinical trials—a practical guide with flowcharts. BMC Med Res Methodol.

[61] Laerd Statistics. Two-way mixed ANOVA using SPSS Statistics. Statistical tutorials and software guides. 2015. https://statistics.laerd.com/.

[62] Kohrt BA (2018). The role of communities in mental health care in low- and middle-income countries: a meta-review of components and competencies. Int J Environ Res Public Health.

[63] Patel V (2016). Addressing the burden of mental, neurological, and substance use disorders: key messages from disease control priorities. Lancet.

[64] WHO (2022). WHO guidelines on parenting interventions to prevent maltreatment and enhance parent–child relationships with children aged 0–17 years.

[65] Grossman M (2020). Youth resilience to violent extremism: development and validation of the brave measure. Terror Polit Violence.

